# Supplementary material for: TDP-43 maximizes nerve conduction velocity by repressing a cryptic exon for paranodal junction assembly in Schwann cells
Source: eLife. 2021 Mar 10;10:e64456. doi: 10.7554/eLife.64456 (PMC7946431; doi:10.7554/eLife.64456)
Supplement: Supplementary file 1. — The exon information was compiled from the current Ensembl database. The coordinates of each exon in chromosome 1 are shown (GRCm38.p6). Transcription starts from either exon 1a or 1b and terminates at either exon 23T or 33. The alternative exons specific for NF155 (exons 23 or 23L and 24) and the ones for NF186 (exons 28–30) are shaded in blue. Other alternative exons are shaded in gray. The cryptic exon CE1 identified in this study is shaded in pink. The start codon and potential stop codons are framed in red. [file elife-64456-supp1.docx]

**Supplementary File 1. The exon nomenclature of *Nfasc* used in this study.**

The exon information was compiled from the current Ensembl database. The coordinates of each exon in chromosome 1 are shown (GRCm38.p6). Transcription starts from either exon 1a or 1b and terminates at either exon 23T or 33. The alternative exons specific for NF155 (exons 23 or 23L and 24) and the ones for NF186 (exons 28-30) are shaded in blue. Other alternative exons are shaded in grey. The cryptic exon CE1 identified in this study is shaded in pink. The start codon and potential stop codons are framed in red.

| **No.** | **Exon** | **Start** | **End** | **Start Phase** | **End Phase** | **Length** | **Sequence** |
| --- | --- | --- | --- | --- | --- | --- | --- |
| 1a | ENSMUSE00000691157 | 132741797 | 132741671 | - | - | 127 | ACGGGCTAGTCTCTGCCCTAATGCGGCGGCTGGCAGCGAGAGGTGCTGCAGGGGACGCGAGGGAAGTGGCGGCGCCGGGAGCGGACATCTGGGACTGCGCCCGGGGCTGGAGCCCGAGCTCCTCGAG |
| 1b | ENSMUSE00001334464 | 132707406 | 132707099 | - | - | 308 | AACTCTCACAGGACGTGGTGGGCTTGAGATAATAAAGCCCTGTTTATGTGACACAGGGCTGGAAGTGACAAGTGGGAGCTGGCGGGGAGCAGGGGCACTCGGGAATGACTTCGGTTCCTCCTCTGGTAGGGCTGCTGTCTTCGCTGTTTAGAACGGCAGCGCTCAGGGGAGAGGATCGAGCTGGGATTTTACTCAGGCTTCGTTTTGCTTACTCGGATGGCCTAGGGCCTAAGAAAGGTCAGCTTTCAGCATCCACGTGTCCCTAAGGAAGGCACCCACCAGTGCAGACTCTGGACAAGCCAGGAGAG |
| 2 | ENSMUSE00000691155 | 132665587 | 132665479 | - | - | 109 | GTTGACTGACTTATGTGCGCTTTGGCAGCCTGGAGTTTTCTTGCCCTCTGCCGACGGGAGCAGAGCCTCTGCTGGTGTTGGGAAAAGCAGGCCGAATGAGGCAGAGAAG |
| 3 | ENSMUSE00000419313 | 132642858 | 132642678 | - | 1 | 181 | CTGAGCACTGTCTGGAAAGCCCACATAGAGCGGCACAAGGCATCGGGAGCCCGTGGGCTGGGCAGCGGGGAGCAGGGCCTGGTGCTGAGGATGGCCAGGCAGCAGGCGCCACCCTGGGTCCACATAGCCCTCATCCTCTTCCTCCTCAGCCTCGGAGGGGCCATCGAGATTCCGATGGACC |
| 4 | ENSMUSE00000967840 | 132637881 | 132637864 | 1 | 1 | 18 | CAAGCATTCAGAATGAGC |
| 5 | ENSMUSE00000242498 | 132637091 | 132636986 | 1 | 2 | 106 | TGACCCAACCCCCAACTATCACCAAGCAGTCCGTGAAGGACCACATCGTGGACCCTCGAGATAACATCCTGATTGAATGTGAAGCTAAAGGCAACCCCGCCCCCAG |
| 6 | ENSMUSE00000159135 | 132634820 | 132634624 | 2 | 1 | 197 | TTTTCACTGGACTCGCAACAGCAGATTCTTCAACATTGCCAAGGACCCACGGGTGTCCATGAGGAGGAGATCTGGGACCTTGGTGATCGACTTCCGCAGTGGTGGGCGGCCTGAGGAATACGAAGGGGAGTACCAGTGCTTTGCCCGGAACAAATTTGGCACTGCACTTAGCAACCGCATCCGCCTGCAGGTGTCCA |
| 7 | ENSMUSE00000159143 | 132634290 | 132634168 | 1 | 1 | 123 | AATCTCCCCTGTGGCCCAAGGAAAACCTAGACCCCGTCGTGGTTCAAGAGGGTGCCCCCTTGACACTACAGTGCAACCCCCCACCCGGCCTCCCGTCCCCCGTCATCTTCTGGATGAGCAGCT |
| 8 | ENSMUSE00000159132 | 132631855 | 132631685 | 1 | 1 | 171 | CCATGGAGCCCATCACCCAGGACAAGCGTGTCTCCCAGGGTCACAACGGGGACCTGTACTTCTCCAACGTCATGCTGCAGGACATGCAGACCGACTACAGCTGCAACGCGCGCTTTCACTTCACCCACACCATTCAGCAGAAGAACCCCTTCACCCTCAAGGTCCTCACCA |
| 9 | ENSMUSE00000914025 | 132627922 | 132627872 | 1 | 1 | 51 | ACAACCCCTATAATGACTCGTCCTTAAGAAACCACCCTGACATATATAGTG |
| 10 | ENSMUSE00000418478 | 132621623 | 132621512 | 1 | 2 | 112 | CCCGAGGAGTTGCAGAAAGAACGCCCAGCTTCATGTATCCCCAGGGCACATCGAGCAGTCAGATGGTTCTCCGTGGCATGGACCTGCTGCTGGAATGCATTGCCTCTGGCGT |
| 11 | ENSMUSE00000159125 | 132621109 | 132620925 | 2 | 1 | 185 | CCCAACACCAGACATTGCATGGTACAAGAAAGGTGGGGACCTCCCATCTAACAAGGCCAAGTTCGAGAACTTTAATAAGGCTCTGCGCATCACCAATGTCTCTGAAGAGGACTCTGGGGAGTATTTCTGCCTGGCCTCCAACAAGATGGGCAGCATCCGGCACACGATCTCGGTGAGAGTAAAGG |
| 12 | ENSMUSE00000159129 | 132619520 | 132619389 | 1 | 1 | 132 | CTGCTCCATACTGGCTGGATGAGCCCAAGAACCTGATCCTGGCTCCTGGGGAAGATGGGAGGCTGGTATGCCGAGCCAATGGGAACCCGAAGCCGACCGTGCAGTGGATGGTGAATGGAGAGCCTTTACAAT |
| 13 | ENSMUSE00000159122 | 132611673 | 132611530 | 1 | 1 | 144 | CGGCACCACCCAATCCCAACCGTGAGGTAGCTGGAGACACTATCATCTTCCGGGATACTCAGATCAGCAGCAGGGCAGTGTACCAATGTAATACATCCAATGAACATGGCTACCTGCTGGCCAATGCCTTCGTCAGCGTGTTAG |
| 14 | ENSMUSE00000159115 | 132610886 | 132610775 | 1 | 2 | 112 | ATGTACCCCCTCGGATGCTGTCTGCCCGCAACCAGCTCATCAGGGTGATCCTTTATAACCGGACACGGCTGGACTGTCCGTTCTTTGGGTCTCCCATCCCAACACTCCGATG |
| 15 | ENSMUSE00000159117 | 132610477 | 132610311 | 2 | 1 | 167 | GTTTAAGAATGGGCAAGGAAGCAACCTGGATGGCGGTAACTACCACGTCTACGAAAACGGCAGTCTAGAAATCAAGATGATTCGCAAAGAGGACCAAGGCATCTACACCTGTGTGGCCACCAACATCCTGGGCAAAGCCGAAAATCAAGTCCGCCTGGAGGTCAAAG |
| 16 | ENSMUSE00000242810 | 132609904 | 132609757 | 1 | 2 | 148 | ACCCCACCAGGATCTACAGGATGCCCGAGGACCAGGTGGCCAAGAGGGGCACCACGGTGCAGCTGGAGTGCCGCGTGAAACATGACCCCTCCTTGAAGCTCACAGTCTCCTGGCTGAAGGACGATGAGCCACTCTACATTGGAAACAG |
| 17 | ENSMUSE00000242804 | 132608507 | 132608383 | 2 | 1 | 125 | GATGAAGAAGGAAGATGACTCCCTGACGATCTTCGGAGTGGCAGAGCGGGACCAGGGCAGTTACACGTGTATGGCCAGCACCGAGCTGGACCAGGACCTGGCAAAGGCCTACCTCACTGTTCTAG |
| CE1 | n/a | 132608090 | 132607987 | 1 | - | 104 | CACCTTCATACCACCAAGTTGACTAAAGCCATCTTGAAGCAATGCTAGACCAAGAGGAGGATGATGAGGACGAGGATGATGATGATGATGATGATGATGATGAT |
| 18 | ENSMUSE00000242616 | 132607047 | 132607003 | 1 | 1 | 45 | CTGATCAGGCCACTCCAACTAACCGTTTGGCTGCCCTACCCAAAG |
| 19 | ENSMUSE00000159142 | 132605585 | 132605484 | 1 | 1 | 102 | GGCGACCAGACCGACCCAGGGACCTGGAGCTCACTGACCTGGCTGAGAGGAGTGTGAGGCTGACCTGGATCCCAGGGGATGACAACAACAGCCCTATCACAG |
| 20 | ENSMUSE00000159119 | 132605238 | 132605041 | 1 | 1 | 198 | ACTACGTCGTTCAGTTTGAAGAGGACCAGTTCCAACCAGGGGTGTGGCATGACCACTCCAGGTTCCCAGGCAGCGTCAACTCAGCCGTCCTCCATCTGTCCCCATATGTCAACTACCAATTCAGAGTCATCGCTGTCAACGAGGTTGGGAGCAGCCACCCCAGCCTTCCATCCGAGCGGTACCGAACCAGTGGGGCAC |
| 21 | ENSMUSE00000159113 | 132603868 | 132603798 | 1 | 0 | 71 | CCCCTGAATCTAATCCCAGTGATGTGAAGGGCGAAGGGACAAGAAAGAACAATATGGAGATCACGTGGACG |
| 22 | ENSMUSE00000159120 | 132602193 | 132601971 | 0 | 1 | 223 | CCTATGAATGCTACCTCTGCCTTTGGCCCCAACCTACGCTACATTGTCAAGTGGCGACGGAGAGAGACCCGAGAGACTTGGAACAATGTCACAGTGTGGGGCTCTCGCTACGTGGTGGGGCAGACGCCTGTCTACGTTCCCTATGAGATCCGAGTCCAGGCTGAAAATGACTTTGGGAAAGGCCCCGAGCCTGACACCATCATTGGGTACTCCGGAGAAGATT |
| 23 | ENSMUSE00000596782 | 132600214 | 132600099 | 1 | 0 | 116 | ATCCCAGGGCTGCGCCCACTGAAGTTAAAATCCGAGTCCTGAACAGCACAGCCATCAGCCTTCAGTGGAACCGAGTCTACTCTGACACGGTCCAGGGCCAGCTCAGAGAGTATCGA |
| 23L | Our unpublished observation | 132600214 | 132600072 | 1 | 0 | 143 | ATCCCAGGGCTGCGCCCACTGAAGTTAAAATCCGAGTCCTGAACAGCACAGCCATCAGCCTTCAGTGGAACCGAGTCTACTCTGACACGGTCCAGGGCCAGCTCAGAGAGTATCGAGTGAGAAGGCTAAGCACCCAGCCCCAC |
| 23T | ENSMUSE00001323414 | 132600214 | 132599835 | 1 | - | 380 | ATCCCAGGGCTGCGCCCACTGAAGTTAAAATCCGAGTCCTGAACAGCACAGCCATCAGCCTTCAGTGGAACCGAGTCTACTCTGACACGGTCCAGGGCCAGCTCAGAGAGTATCGAGTGAGAAGGCTAAGCACCCAGCCCCACGTAACCACCTTCTTGCTAACCAGGGAAAGGGGTAGCAGCCGTGGTGTGTGTGTGTGTGGGGGGGGGGGTGTCTCCAGGGACCCTCGGAGTACTTGTGGGATCTAAAGTTGATTCAAGATGATTTCTGGGATTTGAAATTACCCAGTGGGAAGCATATACATGCATACAAATACTTATGTACATTATACATATGTTCATATATCACTGAACAACAGGAATATGTTCTAAGAAATGTAT |
| 24 | ENSMUSE00000596781 | 132597969 | 132597765 | 0 | 1 | 205 | GCTTACTACTGGAGGGAAAGCAGTTTGCTGAAGAACCTGTGGGTGTCTCAGAAGAGACAGCAGGCCAGCTTCCCTGGTGACCGTCCCCGGGGCGTGGTGGCCCGCCTGTTCCCCTACAGTAACTACAAGCTGGAGATGGTGGTGGTCAATGGGAGAGGTGACGGGCCTCGAAGTGAAACCAAGGAATTCACCACCCCAGAAGGAG |
| 25 | ENSMUSE00000159133 | 132596648 | 132596526 | 1 | 1 | 123 | TACCCAGTGCCCCCAGGCGGTTCAGAGTCCGACAGCCCAACCTGGAGACCATCAACCTGGAGTGGGACCACCCAGAGCACCCCAACGGAATCCTGATTGGATACATCCTCAGATACGTGCCCT |
| 26 | ENSMUSE00000596780 | 132595657 | 132595484 | 1 | 1 | 174 | TTAATGGAACCAAACTGGGAAAGCAGATGGTGGAAAACTTCTCTCCCAATCAGACCAAGTTCTCTGTGCAGAGAGCAGACCCAGTGTCGCGTTACCGCTTCTCCCTCAGTGCCAGGACACAGGTGGGCTCTGGAGAAGCAGCCACAGAGGAGTCCCCAGCACCTCCAAATGAAG |
| 27 | ENSMUSE00001044646 | 132593365 | 132593351 | 1 | 1 | 15 | CTACTCCAACTGCAG |
| 28 | ENSMUSE00000242607 | 132588310 | 132588074 | 1 | 1 | 237 | CTCCTCCCACGTTGCCCCCGACTACTGTGGGTACCACAGGCCTTGTGAGCAGTACTGATGCTACTGCCCTTGCTGCCACCAGTGAAGCCACAACAGTTCCCATCATTCCAACCGTCGTACCTACCACCGTCGCCACCACCATTGCCACAACTACTACAACCACTGCCGCCACCACCACCACCACCACTACGGAGAGCCCTCCCACTACCACTGCTGGGACTAAGATTCACGAAACCG |
| 29 | ENSMUSE00000242595 | 132584480 | 132584364 | 1 | 1 | 117 | CCCCCGACGAGCAGTCCATTTGGAACGTCACAGTGCTCCCCAACAGTAAATGGGCCAACATCACCTGGAAGCACAATTTCAGGCCTGGAACTGACTTTGTGGTTGAGTACATCGACA |
| 30 | ENSMUSE00000242690 | 132583147 | 132582995 | 1 | 1 | 153 | GCAACCATACGAAAAAAACTGTCCCTGTTAAGGCCCAGGCCCAGCCTATACAGCTGACAGACCTCTTTCCCGGGATGACGTACACGTTGCGGGTGTATTCCCGGGACAACGAGGGCATCAGCAGTACCGTCATCACCTTTATGACCAGTACAG |
| 31 | ENSMUSE00000242682 | 132576417 | 132576286 | 1 | 1 | 132 | CTTACACCAATAACCAGGCAGACATCGCCACCCAGGGCTGGTTCATCGGGCTCATGTGTGCCATTGCCCTTCTGGTGCTGATCCTTCTCATCGTCTGCTTCATCAAGAGGAGTCGAGGTGGCAAGTACCCAG |
| 32 | ENSMUSE00000159137 | 132573839 | 132573770 | 1 | 2 | 70 | TGCGGGAAAAGAAGGATGTCCCCTTGGGTCCTGAAGACCCCAAAGAAGAAGATGGCTCATTTGACTACAG |
| 33 | ENSMUSE00000596776 | 132570651 | 132564690 | 2 | - | 5962 | TGATGAGGACAACAAGCCCCTGCAGGGCAGCCAGACATCTCTGGATGGCACCATCAAGCAGCAGGAGAGCGATGACAGCCTGGTGGACTATGGCGAAGGCGGCGAGGGCCAGTTCAATGAAGATGGCTCCTTTATTGGCCAGTACACTGTCAAAAAGGACAAGGAGGAAACGGAGGGCAATGAGAGCTCAGAGGCCACATCACCAGTCAATGCCATCTATTCCCTTGCCTGACGGAGTATCCCAGGCACGGCACTACTTTGCAAGTAGGAGGGGAGAGGGGAGATGAAGCAACTGCAGACCTACCATAAAGCCACCACCACCTTCAGGGACAAGGGTACAACATGGGGGTCTGCCAAGCTGTGAGGGCCAGTGACCACCCAGCTATCCACAACCCCCTCCTAGTGATGCCCCGTTACCCTTGGGTGCCATTAGATGGGAAAGCTAGAGCTATGCTCATTGCAGGGAGCCCTAGTCCCTTGCCCTGTCTCACGGCCACCCTGAGCGTCCCACCACAATGGCCACCCCGATCTCGGCTCACGATCATTCATTTCTATTGCTTACGATATTTTCATTCTCTCTCTCTCTCTCTCTCTCTCTCTCTCTCTCTGCTTTGTGCATCTTCTCCTTCCAAGTCATTGTCTCCATTTTCTTTTACTTTTGAAAAAGAAAAAAAAAAAGTCCCTGGAAAAAGAATAAGTGGGCTCTCCCCAAGGAAATGAAAACGATGGGCAAAAAAAGGATTGATCCGTAACACAACATGCAGAAAAGCTGTAGTATTCAAGCTGCCACCAGGCCAGTGTGTTTCCGAAGGATGCCTTTCGCCATATGTCTCCCCCAACCCCCACTCCCCACCCATCTGCCTCGGCCACATCCATGCTGAGCTGGGCTTGGCTCTTTTCTGGAAAATGACAGTGTTTTTTGGCAGGAAGAGGTGCGCAGGACTCCTTTCCATGCCCTGCTTCAGTTGGGAGGGGGCCTTACCTCTTGCTCCTGGTTCTTACTCCGCCCCTGCCACCAGAGAGTCTGTGGATGAGCTGCTCGGATGATGAGAAAGGGAGACCTACAAGGACTCTGGCGACCAGACCCGAAGCTGGAAGCAAGTCAGAGTGTGGTCCCTGGATAATGGAGCAAACCTCCAGAGAAGAGAAAGGGCTGGGTTTGGACCTCTTTTGTCTTTGGCAGTGAAACCCAGGGTGGAGGAGAGAGGGCCACACCTGCTCACCTGGTCAGGGTCTATGCTGCTACCCTCCAAGAGCTGGAGCCCTGAGATTCAGGAGTGTGGCTGGTGTGTCTGGGGGTAACAGTGCTCCTCCTCCTCCCGTCCTCTCCTTGCTCAATTGTGTCTGACATTGACTTGTGTTTGACATGGTATATGAATGGGGATCTGCTGCCCCCAGAATGAGCCTATCTGTCCTTTCATTAGGGTACGCATACATATCGGGGGACCGTGAGGTGCACCGTAGTAACAGTGCTGTGATGCCTCTCCCTGCCTTGAAGGACACAAAGCTCTTCGTATGCCTTATACAGCTCGGATCTAACCCTGCGGTTCCCAGATCCCTAGCCCTATTCTGCAAGTCTTGATGCCTCCAGTGATGTGTGTCTCGGGACCAGGGCAGCCTATGCAAGTGTCCCCTCGGGACCAGAAGCAGCCGCTCCACACACGCCCTTACCCCCCCTCCGCCCTAATGGAGACACTCGCAGGCATGCAGTTCTCAGCCTGAAGGTGCTGCCTTCATTCCCCCACCTAACCCACTTTTGAAACCAAAGGTACCTTGCCTCAGCGACACAAAGAGATGGGAGATCTCAAGCCAGAGTGTCGCCCTGTAGCTGTGTCTGCAGCCTGAGGCAGTTGTGGGAGGGCAAGGTTGACCACTTCGAATGGATCGCCCGCCATTGGCATGGCCTCCAACTTGAGGTACTAGTTAGGGGCCTGGGTGGGACCAGGGCTGTGCATTCCAGAGACTTTCCAAGAGCATCATTTGCCATACGTTTAAACCGCAGAGGTATTAACAATGCTGTGTCACTTGATAAGCATTTGGGTGAGCGTCAACCCCGTAGGCACACACTCTTTGCAGGTAAGAAATCATATCCCAGAGATGAGCTGATAATGTTTGAACCTGAGAGTGTGTCATGGTCCTTTGGACCAACCCATGGCCAGCACTGCTTCCTGGAGGCCTGAGGGACAGCTCTCATGGTGAAAGGGGACAGGGTAAACAGGAAGGCTAGGGGTGGGGGTGTGCTCAGGTCCCCTTCCCTGTTCTAGTCTAACACTTCTGGGACACAGCTGGGCATTGCTGTAGTGTAGGTCACCATTCCTGACGGGAGGGTTAGGGAAATGCAACTGAGGTAAAGAACCATCCTTCCCAGAAAACACTGTGGACCGGGAGGAAGTGGTGGGTTTCTCAGGCACCATTTACACAGCACCATCCAGCTAGTAAAGCCATGGCACCAACAGCAGGCATTCAGAGGAAGGGACTCGGTTGTCATTGTAGCTATCCCCCTGCCACAGGCAGCCTGGAGAGGGCTCTTTGGTGTTAAGGGTCTGGGAGAGAGGTGAATCCTTAGGCTGTCTTTGTGCCTCATTCCGGAGTTCTGTCATGCTCAGTACTGTCCTCTACAGAAGGAATGAAAAGCCCATCAGCCCCTCCCTAAGGCTGTTGTGTTGTGGGTGCTGAGAGCTGTCCCAGCCACCAGCGGTCACAGCTCTCCTGGCAGGAACAGATTGGGGGCTGGAGAACCAGAGAAATGGCAGGTTCCCAGACAGAGCAGCTCAGCACTGTGAGGTTTCAAGGAGGCAGCTTTTTCCAAGAAGGGGAGAGGCCACTTTTCACAGAGCCTGCCAGAGTCTACCCTCAACTCAGCAAGAGAACAGTATTGCCACCCCTGCACCTGGATCTTAGGCAGCCACCCCAAAGTGGTGCCTAGGCCTGCTCGTGTTTCATCTCTCAGGTGGGAGCAGGGGGACAGGGTGGGTCATCCTGCAGTGATTCTGAGTGCTTATCAAAAGCTCTTTGAGCATCTAGTCTGGGCCAAAGCTTAAGCCATGAATACCATGTGAACAAGTGAGGTCAGATGTGGTCCCTACCCACGCAGGGCTGATGTTACTGGAAACGAGTAGGTGAGTTCCTGGCACACCGTGACCATTCCGTCAGAAGCGTTGTCCTGGTTTGCCTATACTGCCGATTGCTTCAAGTCAGAAGGGAGAGGTTTCCAAAGGAGAACTTGCTCCATTGGGCCAGCAAGTCTGGGCAGGACATGACGGTCAGGATGAGTGGATTCGGTAGGCAGCACTGTAGTGCTTTAGGGTGTCTGTCTCCCACATTTAGCCCCCTCTGTGAATACTCCTGTTTGGAGGAGGACAGGTGTACCCGATCAGGACCAACTGTCCCCCATCCTGCCTGGGATATGCTAAACAGGACACATCAAGGCTCTACTCGTGTGTAGATAGCTGAGTTAATACTAGAGGGAAAGCCAGGCTGGCTCCTGCCCTTACCTATTTCCTCCTTCCCAGAGGTCAGTGTAGGAGGGACCACCCCTCCCAACAAGTCCCAAGAGTCAGCCAGTGATTCAGCTCACCTCACAAAAGGAACAAGAGAGCACGTTCCTGGTTTCCCTGAACACAGTCAGCATTGAGAAGGGAGTAAAAGCATCCTCCCCATCCCCCATACACACACACACACACACACACAAACACACACACACACAAGCAGACCCAACTGTAGGGAACACCCTAGATCATAATCTTACAGCATGAGCATCCATATCTTGGTTCACATGAGATGAGCTGCTAAAGCCTGCGGGTGAGGGGACCTTGGGGAGGGAGAAGCTGTTCAGATTGGGCCTTATTTTTGGTTCCCGTGGACAGAAGGTAGAGAAGAAATCTGAAGGAGAGAGAAACTGGCCTTTGACTCTCCTGGCCCCTCCTGGGAGCCCCAGCTAAACTCGGCTGTCCTAGAGCCTCTGGATGGGAAGCAAGGTTTCCTTGCTGTTTATGAGGCTTTTTCGTGGTTGCTCAGAACGGATGAAGCTTCTTGTTCCCTCCATGGTCTAGGGCCGAGGCCCAGACTCACTTCTCAGAGGAAGGAGAGCAGGTTGCTAGAATGCCAGCCCAGAACCCGTCCCTCATCTGTGTCAGTTCGGAGTGGGGTGTCCTCCAACCAAGACCAGCAGCCTTTGCCCAATGAAAGCCCATGGCCAGGATTAGGTCCCAACAGCTGGGCCACAGTGCTCTCTTTCTAGTGAAGCAAATGTCCTTACATATATTTCACATGGTAGCCTAGGAATCCCATGTAGGCAGTCCCCACTCCAAAACAAGAAGTCTCAAGCCAGTGACAGAAAATGTAGAGTGTAACACCGGCAGCTTGGGCAAAGAGTCCCCAACTCAGTGGCTAAATGCCTGAGACCAGACCCGTGGCTGTCCCCACCCAACACAGCCTCAGCCCAGAAAGTGCCCTTGCTTTGCTTCCTCTTCTCAGAAGGGGACTCTCAGAGACACATTCAGGAGAGGACAGGGAAGGACACGTGCCCAGGGAAGCTGCAAGACTCCCTGAGCTATCTCCAGCCACGGCGGAAAGCATTGTTGCTCTTATCAGCACATTAGTATTAAAAGGCTGTTGCTTGAGCAGAGATGGAGATGAAACAGACGCAAGGCATCTAGCTCAGGACAGAATCGGATTCCTCATGTGAAGGAACCTCAGAGCTGATCTGTTTAAAATGACTCATCATACAGACAGGGAAATGACAGTGCGGGGAGGGAATCTAGCCAGTGAGGGTACATTTAGGACTTGAACTTGAGCCTTCTGGGCCTTCTGACTCCGTGTCCAGTGTTCTTTCTTCTCACCACAGCTGCCTCAGCAGGTGTTGCCTGAGATCTGTCCTGCTTGTCACCATGTCACCTCTCTCAGCCATTACACTATGTTCCCAGAACCTCCCAGCAAACAACCCAGGCTTTTCCTGAGTCTGAGATCAGCCCGTCTGCGATAACCTGTGATTAGCTCAGTCATGGAGTCCCAGGCAGCCCGAGGCTGGAAGGTAGAAGGATGAGCCCGTAGGTGTTTGCTACTGTGAAACCTGGACTGGGCCCCGGGATGGTGTCTCTTGATTCATCACACGCCTGGCCTTCAGCATACCGTATTCCTCAGAAGGGAGGGTAAGGAGGGGTGTCTCTGTCCATCCTGCCCTTGACCGCCAAGTCTGCAGGTATTGAGGGGTAGCAGGGCCCAGGCAAGTCTCCCACCCTGGGATCTGCCGAGCTGGAGTTTAGCAGATGTAAAGACTTCTATGAAGCAATAAACACAAAAGTCTGGGAGAAGCTATCCAGAGTTTTGTGCAATCCTGTTTCTTTTTCAAAGCTGTAGCAGATCAGGTGACCCTGACCTGCTTGCTCTTCTAACAAATGTGATGTCTTCAGTCAGTGTTTGCCTCTACCCGGCTTCCCAGCTCTTTGCCAACTTCTTCTGAGCTGAGTTATCAGTCGCCTGTGACACGCCCATTCTCTGTCCTGGTCCCGCCCCCAACCATGGTGGATCCTGGAGGACCTCGTACCCCTCCCCTCCCCCACATATGCATCTCTCCCTTCCGACATGGTGTTCCCTGTGTCTCCATGCTGCCCACATTCCTAAGCACATGAGTTGGTCTAATCTTTTTTTCCATTGGTTACCTTTAACTCTCCATGGCAGGCTCACTTTAATCCCTCACATCATGTCCTTCCCTTCTTGTGAGTTATTTGTCATTAACCGACATTGTCAGCGACAGACGTGTATCTGAGGGTGTCATGCACGACCTTCAGCAGGGACTTCTGGGCCATGGAGGACTGTCTAATACATGGACTTATAAACTGGCTGCATGAGCAATGAAAAGGCCAAATTATTCTGATTTTTTTTTTGAATCACTGTAAAAAAAAAAGCTGATTTCTTTTGTATAGAGAACACTAAACGTATAATAAAAGTTGTTCAAAATGGA |
|  |  |  |  |  |  |  |  |
